# Comparative transcriptome analysis linked to key volatiles reveals molecular mechanisms of aroma compound biosynthesis in *Prunus mume*

**DOI:** 10.1186/s12870-022-03779-3

**Published:** 2022-08-09

**Authors:** Wang Xiujun, Song Zhenqi, Ti Yujing, Ma Kaifeng, Li Qingwei

**Affiliations:** Beijing Key Laboratory of Ornamental Plants Germplasm Innovation and Molecular Breeding, National Engineering Research Center for Floriculture, School of Landscape Architecture, Beijing Forestry University, Beijing, China

**Keywords:** *Prunus mume*, Floral scent, Transcriptome, Transcription factors, Gene expression and regulation

## Abstract

**Background:**

Mei (*Prunus mume*) is the only woody plant in the genus *Prunus* with a floral fragrance, but the underlying mechanisms of aroma compound biosynthesis are unclear despite being a matter of considerable interest.

**Results:**

The volatile contents of the petals of two cultivars with significantly different aromas, *Prunus mume* ‘Xiao Lve’ and *Prunus mume* ‘Xiangxue Gongfen’, were characterised by GC-MS at different flowering periods, and a total of 44 volatile compounds were detected. Among these, the main substances forming the typical aroma of *P. mume* were identified as eugenol, cinnamyl acetate, hexyl acetate and benzyl acetate, with variations in their relative concentrations leading to sensory differences in the aroma of the two cultivars. We compiled a transcriptome database at key stages of floral fragrance formation in the two cultivars and used it in combination with differential analysis of floral volatiles to construct a regulatory network for the biosynthesis of key aroma compounds. The results indicated that *PmPAL* enzymes and *PmMYB4* transcription factors play important roles in regulating the accumulation of key biosynthetic precursors to these compounds. Cytochrome P450s and short-chain dehydrogenases/reductases might also influence the biosynthesis of benzyl acetate by regulating production of key precursors such as benzaldehyde and benzyl alcohol. Furthermore, by analogy to genes with verified functions in *Arabidopsis*, we predicted that three *PmCAD* genes, two *4CL* genes, three *CCR* genes and two *IGS* genes all make important contributions to the synthesis of cinnamyl acetate and eugenol in *P. mume*. This analysis also suggested that the downstream genes *PmBGLU18-like*, *PmUGT71A16* and *PmUGT73C6* participate in regulation of the matrix-bound and volatile states of *P. mume* aroma compounds.

**Conclusions:**

These findings present potential new anchor points for further exploration of floral aroma compound biosynthesis pathways in *P. mume*, and provide new insights into aroma induction and regulation mechanisms in woody plants.

**Supplementary Information:**

The online version contains supplementary material available at 10.1186/s12870-022-03779-3.

## Background

The aroma released by flowers is an important aspect of the quality of ornamental plants [1], and arises from the presence of floral volatile organic compounds (VOCs). These are plant secondary metabolites [2], mainly comprising aromatic compounds, terpenes, fatty acid derivatives and other volatile compounds with low molecular weight [3]. To date, over 1700 VOCs have been identified across more than 90 plant families [4]. These compounds play valuable roles in attracting pollinators and as a defence mechanism against other animals and microorganisms [3, 5], and can also improve the aesthetic value of ornamental plants, benefit human health, and serve as integral components of cosmetics, fragrances and condiments [6, 7]. However, the aroma originally typical of *Prunus mume* was lost when enhancing its resistance to cold by crossing with closely related species of plum and apricot that are tolerant to such conditions. As a result, the floral fragrance of *P. mume* has been explored frequently [8], with recent research focusing on temporal and spatial changes of *P. mume* aroma compounds, and the molecular regulatory mechanisms of these VOCs [9–11].

The species *P. mume*, commonly known as the Chinese plum, is a traditional ornamental plant of the genus *Prunus* (*Rosaceae*) that flowers in early spring in China. It is also an important fruit tree with high economic value in China and other Southeast Asian countries, and is the only known member of *Prunus* that can send out a strong floral fragrance [12, 13]. Whereas there are considerable variations in aroma type and composition between different cultivars, the typical aroma substances of *P. mume* seem to be eugenol, benzyl acetate and hexyl acetate [14]. Current studies appear to focus mainly on the identification of floral aroma components, together with their dynamic temporal and spatial changes [15, 16]. For example, the biosynthetic pathways for benzyl acetate and eugenol both belong to the broader phenylpropene metabolic pathway, which has been better characterised in the model plants petunia and *Arabidopsis* [17, 18]—with the synthesis of benzyl acetate catalysed by benzyl alcohol acetyltransferase (BEAT), using benzyl alcohol and acetyl CoA as substrates [19].

**Fig. 1:**
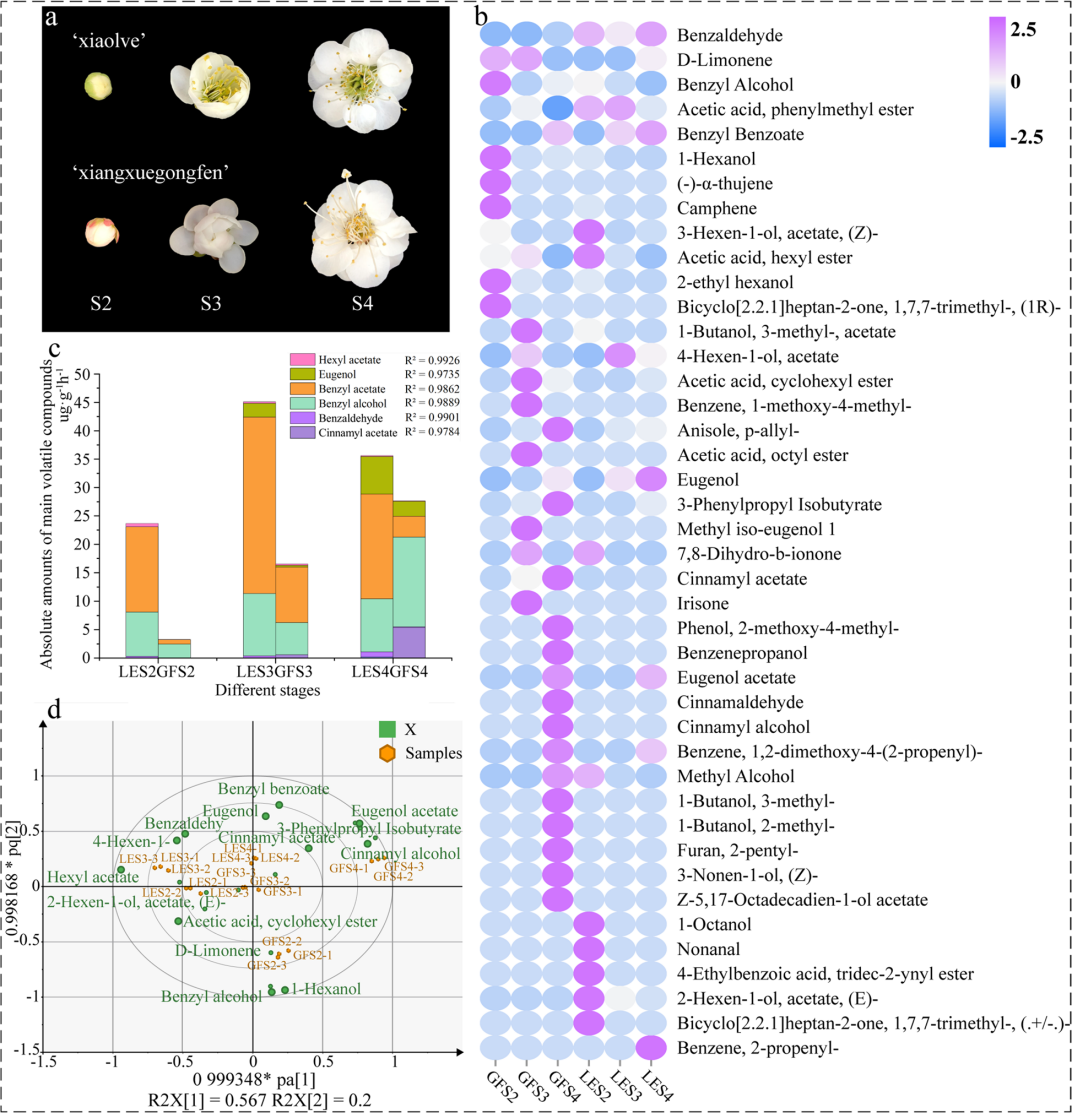
**a** Different developmental stages of *P. mume* ‘Xiao lve’ (LE) and *P. mume* ‘Xiangxuegongfen’ (GF) flowers: (S2) petal baring period; (S3) early flowering period; (S4) full flowering period. **b** Heatmap analysis of volatile compounds of two *P. mume* cultivars at three flowering development stages. The colour of the heatmap ranges from dark blue (value, − 2.5) to magenta (value, + 2.5) on a natural logarithmic scale. **c** Amounts of the six main floral aroma volatile compounds during three different flowering stages of GF and LE. **d** Score plot of OPLS-DA model of headspace volatiles of *P. mume* cultivars with the statistical parameters: R^2^X = 0.999, R^2^Y = 0.994, Q^2^ = 0.967

Although some studies have addressed the molecular mechanisms regulating flower fragrance in different *P. mume* cultivars [20], few of them have focused on the differences in floral VOCs between cultivars [21, 22]. Furthermore, to the best of our knowledge, there are currently no reports that compare and analyse the transcriptome of different *P. mume* cultivars at different flowering stages. We therefore considered it important to investigate the diversity of volatile components in different *P. mume* cultivars, as well comparing differences in aroma composition using headspace volatiles and endogenous extracts [23]. To clearly elucidate the molecular mechanisms of VOC biosynthesis and compare them between cultivars, combined transcriptomic and metabolomic analysis was adopted since this has been established as an effective method and has been applied in many plants, including *Chimonanthus praecox* [24] and *Nymphaea colorata* [25]. For sample collection in this study, petals were harvested from *P. mume* ‘Xiao Lve’ (LE) and *P. mume* ‘Xiangxue Gongfen’ (GF) at three developmental stages (Fig. 1a) and analysed by headspace solid-phase micro-extraction (HS-SPME), combined with gas chromatography–mass spectrometry (GC-MS) for headspace volatiles and endogenous extracts. Differential compounds in the two aroma types were then screened as biomarkers using variable important for prediction (VIP) scores. RNA-Seq analysis was performed on petals collected at the three developmental stages to permit screening for differentially expressed genes associated with floral aroma synthesis. In addition, further analysis using odour activity values (OAVs) was able to screen out several biomarkers that make significant contributions to the two aroma types. Ultimately, our comparative molecular-level analysis of the mechanisms underlying differential expression of aroma-related components of the two cultivars—and our search for the synthetic pathways of aroma compounds in *P. mume*—aimed to provide a theoretical basis for molecular breeding to achieve specific *Prunus* floral aromas, and to enhance essential oil production.

## Results

### Major floral aroma constituents and qualitative and quantitative analysis of the *P. mume* cultivars

In total, 36 compounds were detected over the three developmental stages of GF, including 12 esters, nine alcohols, four phenols, three terpenoids and two aldehydes. For LE, a total of 28 compounds were detected, including 12 esters, six alcohols, three terpenoids, two aldehydes and two phenols. Of the compounds identified, 15 were unique to GF, and seven to LE (Fig. 1b and Table S1). The percentage of each aroma compound in the total volatile substances released differed between the two *P. mume* cultivars (Fig. 1c). An OPLS-DA model was developed based on the relative contents of headspace volatiles in the two *P. mume* cultivars at the three developmental stages, which enabled identification of the differential biomarkers in headspace volatiles of the two cultivars, and the cross-validated predictive capability (Q^2^ = 0.967) manifested the feasibility of the model (Fig. 1d). Among the VOCs detected, cinnamyl acetate, eugenol, benzyl alcohol, benzaldehyde and benzyl benzoate varied greatly in content between the two cultivars across all flowering development stages, which may be an important factor underlying their differences in floral fragrance. Together with the OAV analysis (Table 1), this confirmed that among the shared aroma compounds, benzyl acetate, eugenol, benzaldehyde, benzyl alcohol and hexyl acetate were present at relatively high levels, consistent with previous studies on the characteristic aroma compounds of *P. mume* [26].

**Table 1:** Odour description and OAVs of the major compounds detected in *Prunus mume* samples

Compound	Odour description	Threshold (μg/kg)	OAV					
			LES2	LES3	LES4	GFS2	GFS3	GFS4
Eugenol	spicy, smoky	6	0	3114	8557	0	416	3410
Benzyl acetate	fresh floral, sweet, fruity	30	3749	7781	4620	198	2436	920
Benzyl alcohol	sweet, floral, fruity, cherry, fatty	100	605	849	722	191	439	1218
Benzaldehyde	fruity, almond, burnt sugar	320	7.3	8	20	0	0	3
Hexyl acetate	sweet, green fragrant, fruity	115	39	20	11	1	22	6

### RNA-Seq analysis of developing flowers of two *P. mume* cultivars with different floral aromas

To explore the molecular mechanisms underlying the formation of different fragrances between LE and GF, petal samples of the two cultivars collected at the S2, S3 and S4 developmental stages were subjected to RNA-Seq analysis, with each sample run in triplicate (*N* = 3). Therefore, a total of 18 libraries were constructed and analysed, yielding 58.49 GB of clean reads. More than 97% of bases were above the Q20 threshold, and the GC content was above 46% (Table S2), indicating a high overall sequencing quality.

**Fig. 2:**
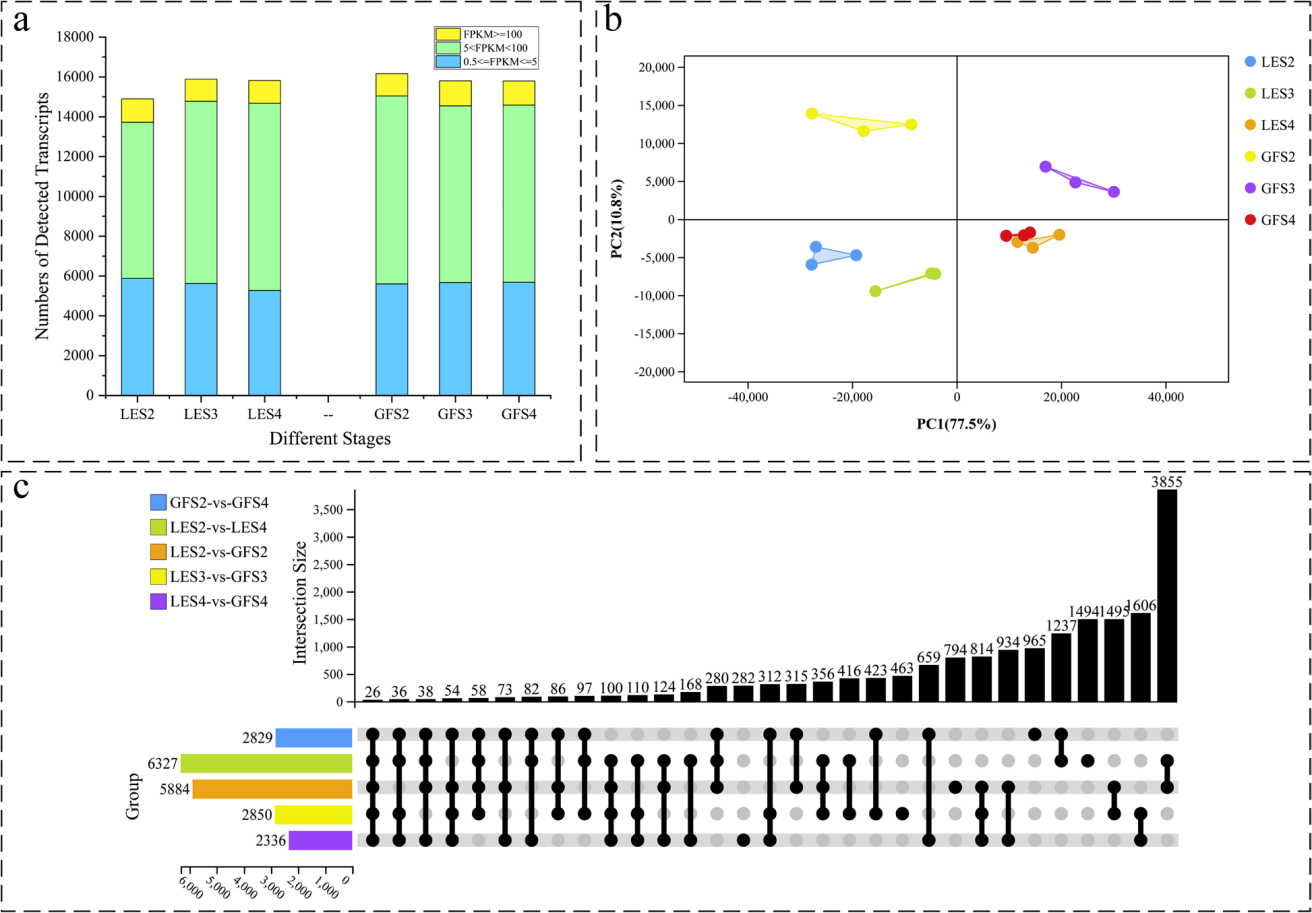
**a** Number of detected transcripts in each sample. **b** Principal component analysis of RNA-Seq data. The PC1 coordinate represents the first principal component, and the percentage in brackets represents the level of contribution of the first principal component to the sample difference. Similarly, the PC2 coordinate represents the second principal component, with the percentage in brackets indicating the value of the contribution of this principal component to the sample difference. The coloured points correspond to distinct samples, as indicated in the legend. **c** Venn diagram of differentially expressed transcripts at the three flowering stages of GF and LE

The number of transcripts expressed in each sample, after genes with normalised reads with FPKM < 0.5 were removed, is shown in Fig. 2a. In total, 16,160, 15,804 and 15,796 transcripts were detected at the S2, S3 and S4 flowering stages, respectively, of GF. Similarly, 14,900, 15,894 and 15,857 transcripts were identified in the equivalent samples for LE. Approximately 36% of the expressed genes were in the range of 0.5–5 FPKM, and 57% were in the range of 5–100 FPKM. The RNA-Seq data were uploaded to the Sequence Read Archive (SRA) of the National Center for Biotechnology Information (BioProject ID: PRJNA786063) for access by the scientific community.

To investigate global differences in transcriptome dynamics during flowering in LE and GF, principal component analysis (PCA) was carried out on the RNA-Seq data obtained for the samples taken at different periods (Fig. 2b), which could be clearly divided into three groups corresponding to S2, S3 and S4. The data for S2 and S3 clustered significantly differently between LE and GF, indicating that these flowering stages differed considerably in their transcriptional programmes between cultivars. The closer grouping of S2 and S3 for LE than for GF indicated that these two developmental periods were more closely transcriptionally related for the former. For stage S4, the data for LE and GF clustered together, indicating a high similarity in their transcriptional programmes at this stage; thus, the greater transcriptional divergence observed at S2 and S3 may explain the different floral scents of the two *P. mume* cultivars.

### Identification of differentially expressed genes, and enrichment analysis

Pairwise comparisons were made for GF and LE at each developmental stage. In each comparison, differentially expressed genes (DEGs) were screened for by expression level with the constraints FPKM > 5, FDR < 0.01, and log_2_ fold change > 1 or < − 1. The results showed that 5884, 2850 and 2336 genes were differentially expressed between LE and GF at stages S2, S3 and S4, respectively (Fig. 2c). In order to screen further for genes related to flower aroma formation, pairwise comparisons were made between the two important flowering stages, S2 and S4, and identified 2829 and 6327 DEGs between these stages for GF and LE, respectively.

**Fig. 3:**
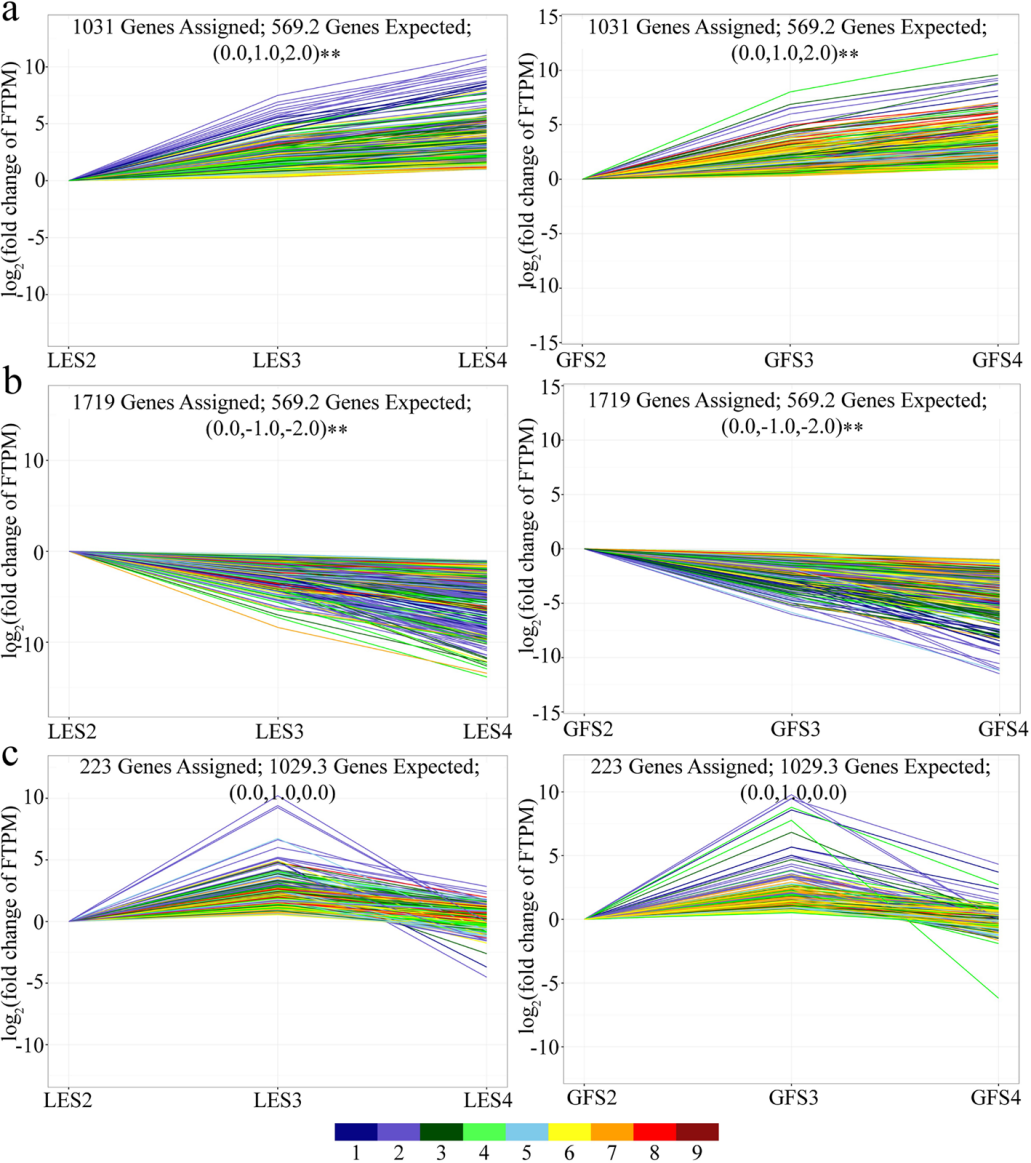
Unigene expression profiles during flowering of two *P. mume* cultivars. Three expression profiles were selected by gene screening: **a** profile7 (group I; top row), **b** profile0 (group II; middle row) and **c** profile5 (group III; bottom row), where profiles 7 and 0 denote upregulated and downregulated unigenes, respectively, and profile 5 denotes upregulated (from S2 to S3) then downregulated (from S3 to S4) unigenes. The nine different coloured lines denote absolute expression levels over the three flowering periods, with FPKM values of 0–0.1, 0.1–0.7, 0.7–2, 2–4, 4–8, 8–20, 20–100, 100–1000 and 1000–12,500 indicated successively by colours 1–9

According to their expression profiles, 2973 of the DEGs were assigned to one of three groups (I, II, III), consisting of 1031, 1719 and 223 individual genes, respectively. The first two groups contained genes that were upregulated (group I) or downregulated (group II) as the flowering stages progressed, whereas the genes in group III were upregulated between S2 and S3 but then downregulated between S3 and S4 (Fig. 3).

In addition, gene ontology (GO) enrichment analysis was performed on the identified DEGs, with the main GO categories shown in Fig. S1. A total of 50 GO annotations were obtained for 8980 DEGs in the three functional categories of biological process, molecular function and cellular component. The main terms (with the associated number of genes in parentheses) included metabolic process (5667), catalytic activity (5310), cellular process (4945), single organisation process (3880), binding (3800), cell (2901) and cell part (2901). The GO annotations of these genes may help in determining the molecular mechanisms of flower aroma production and release.

To identify relevant genes that were significantly enriched in metabolic or signalling pathways, DEGs for each of the S2, S3 and S4 periods were mapped to the KEGG database. For the three flowering periods, 1618, 1095 and 1059 DEGs, respectively, were enriched in the KEGG PATHWAY database. Among these, the metabolic pathways with more annotations of the DEGs at different periods were metabolic pathways, biosynthesis of secondary metabolites, ribosome, biosynthesis of amino acids, plant hormone signal transduction, phenylpropanoid biosynthesis, and pentose and glucuronate interconversions (Fig. S2).

**Fig. 4:**
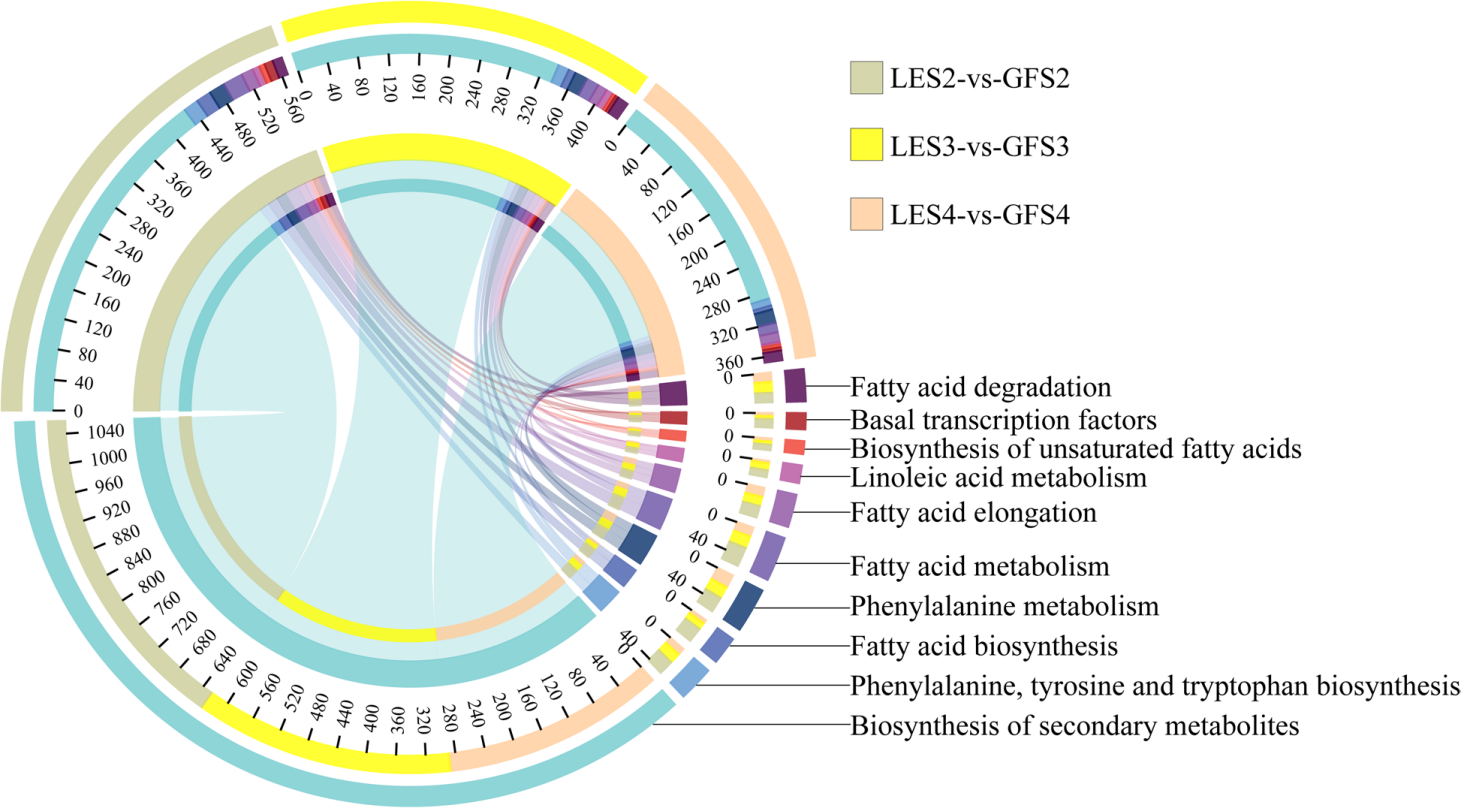
Distribution of DEGs, at different flowering stages of GF and LE, in enrichment pathways associated with major floral aroma compound anabolism

As discussed above, plant aroma constituents mainly include terpenoids, aromatic species and aliphatic compounds. Based on known metabolic pathways related to the synthesis of these three substance classes, 739, 614 and 504 DEGs—related to 13 metabolic pathways—were identified at the S2, S3 and S4 periods, respectively, of GF and LE (Fig. 4). For the three flowering periods, metabolic pathways associated with the biosynthesis of aroma compounds that were more extensively annotated with DEGs were: biosynthesis of secondary metabolites; phenylpropanoid biosynthesis; fatty acid metabolism; and phenylalanine, tyrosine and tryptophan biosynthesis.

### Identification of DEGs related to regulation of flower aroma metabolism at different developmental stages

**Fig. 5:**
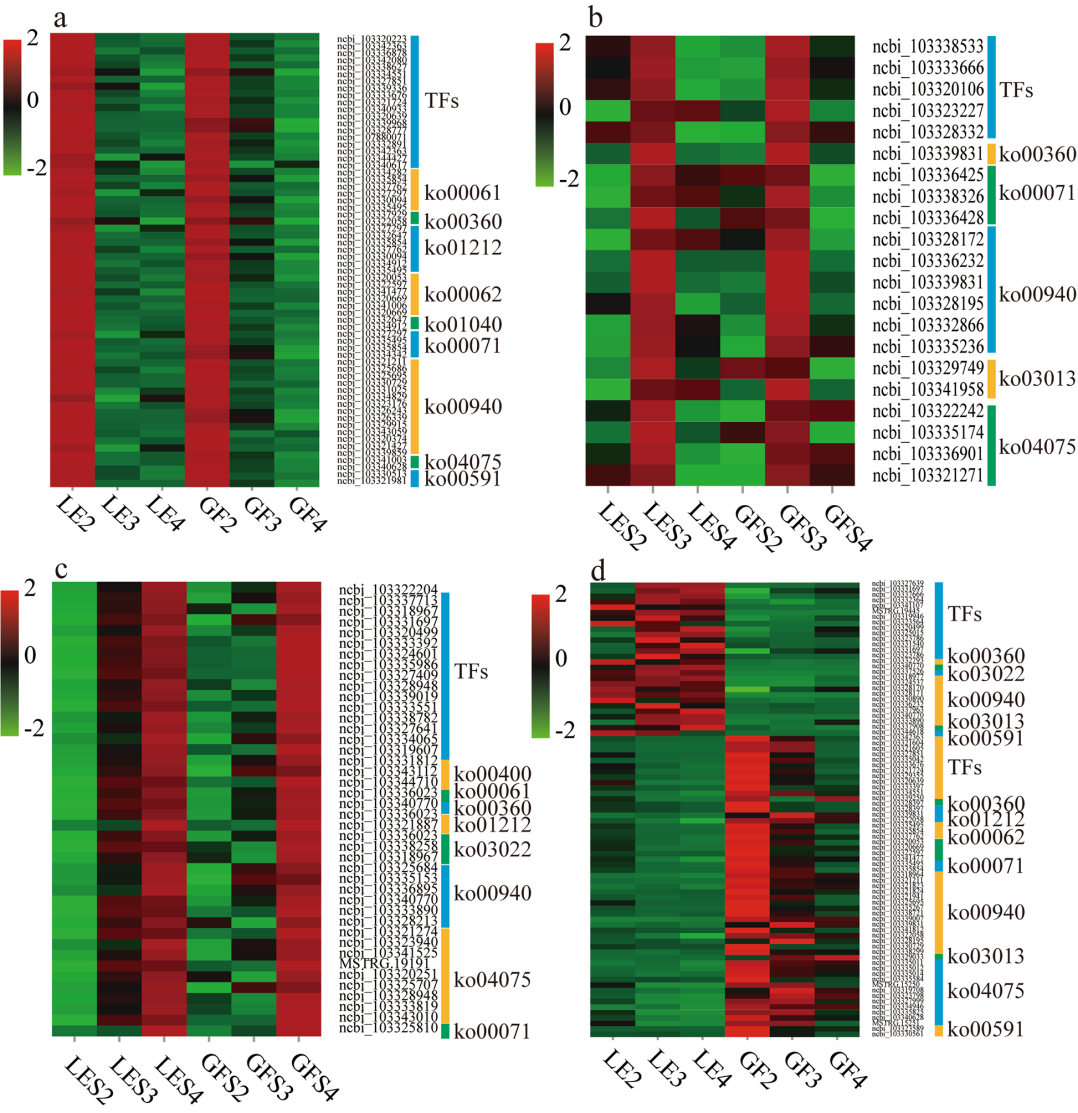
Heatmap plot of DEGs identified by enrichment analysis at the different flowering stages, showing functional categories of significantly over-represented DEGs at S2 (**a**), S3 (**b**), S4 (**c**); and in LE vs. GF (**d**). The name of the pathway associated with each KEGG term are in Supplementary Table 4. The colour of the heatmap ranges from green (value, − 2) to red (value, + 2) on a natural logarithmic scale

More detailed comparisons were then made between the three flowering periods of GF and LE. Genes that were significantly differentially expressed between cultivars at S2 were further screened by comparison with S3 and S4 (Table S3), and the pathway classification of DEGs enriched at S2 is shown in Fig. 5a. At this flowering stage, a large number of transcription factors (TFs) and genes associated with metabolism of floral volatiles were highly enriched in both cultivars—including protein coding genes for phenylalanine metabolism, fatty acid biosynthesis and phenylpropanoid biosynthesis—suggesting that regulatory networks including phenylpropanoid biosynthesis and fatty acid metabolism control the synthesis of aroma compounds in both *Prunus* varieties. Similar analyses identified genes significantly overexpressed at S3 (Fig. 5b, Table S3) and S4 (Fig. 5c, Table S3); and DEGs that were more highly differentially expressed between cultivars over all three flowering periods were also screened out, then functionally enriched and classified (Fig. 5d, Table S3).

### Construction of gene coexpression networks

**Fig. 6:**
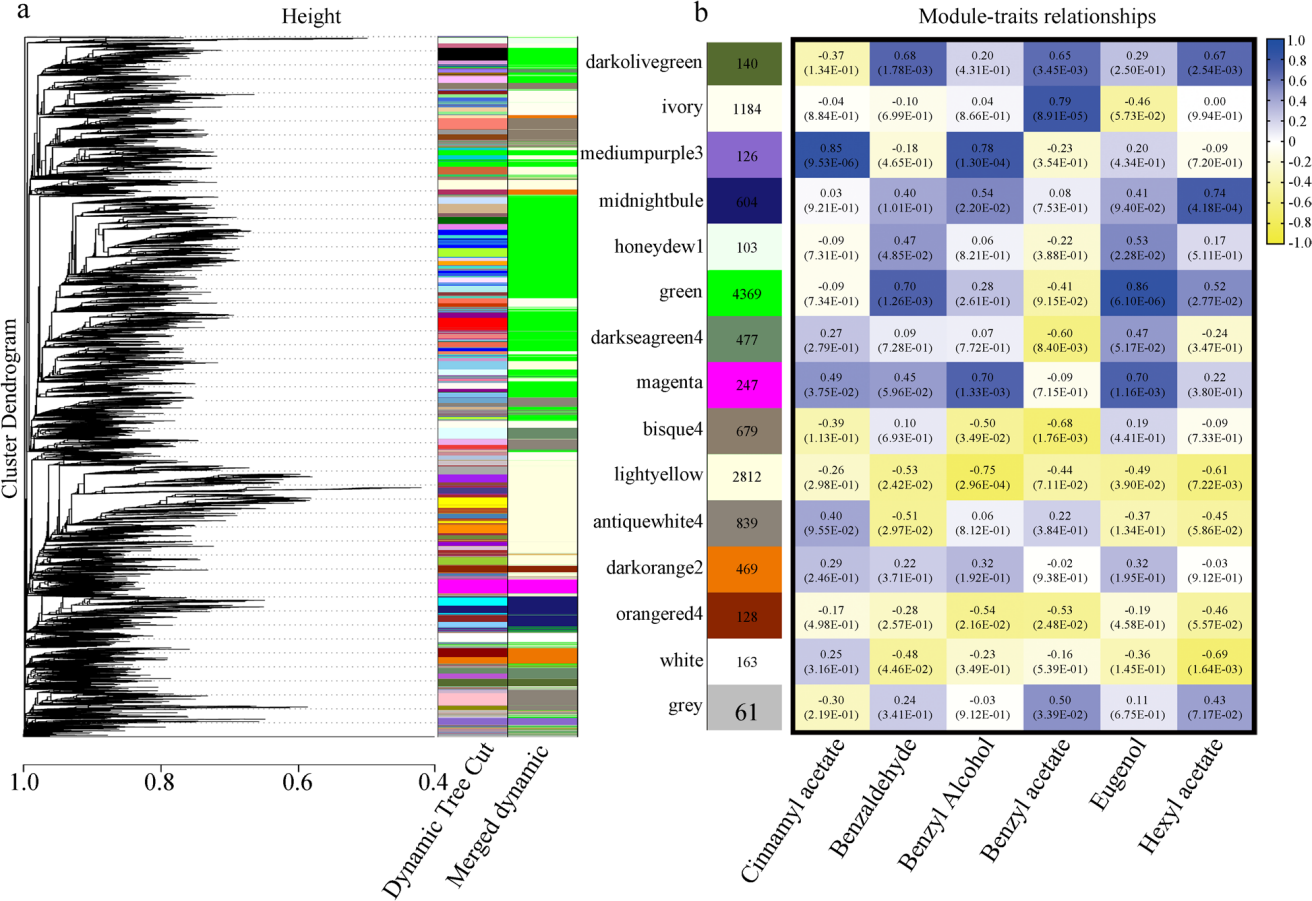
Coexpression network analysis for the three flowering stages of GF and LE. **a** Hierarchical cluster tree showing coexpression modules identified by WGCNA. Each leaf in the tree represents one gene. The major tree branches constitute 15 modules, labelled with different colours. **b** Trait association correlation analysis. Each row corresponds to a module and is marked with the colour corresponding to panel a, with the number of genes in each module shown on the left. Each column corresponds to a specific floral volatile compound. The colour of each module at the row–column intersection indicates the correlation coefficient and *p*-value between the module and the volatiles. The legend (top right) shows a colour scale for module trait correlation, from − 1 to + 1

Weighted gene correlation network analysis (WGCNA) was then carried out in order to gain a comprehensive understanding of the genes expressed in GF and LE at successive developmental stages, and to identify specific genes associated with the synthesis of floral volatiles. After screening out low-expressed genes (FPKM < 0.05), WGCNA retained 12,401 genes. Coexpression networks were constructed based on pairwise correlation of gene expression across all samples. This analysis identified 15 distinct coexpressed modules (Fig. 6a), seven of which (mediumpeople3, bisque4, green, lightyellow, ivory, midnightblue and white) were significantly associated with changes in floral volatile content during flowering (Fig. 6b). More specifically, cinnamyl acetate and benzyl alcohol content were highly positively correlated with mediumpeople3 module gene expression; cinnamyl acetate and benzyl acetate were negatively correlated with bisque4 module gene expression; benzaldehyde and eugenol were positively correlated with green module gene transcript abundance; benzyl acetate, benzaldehyde, benzyl alcohol and eugenol were negatively correlated with the lightyellow module; and benzyl acetate was highly positively correlated with ivory module gene expression. Finally, hexyl acetate was highly positively correlated with the midnightblue module, but negatively correlated with the white module. Based on the correlations between genes of these seven modules and the floral volatiles, together with the KEGG PATHWAY enrichment results for floral aroma compound anabolism (as described above), we identified a total of 957 structural genes potentially associated with the biosynthesis of floral volatiles. The expression patterns of these genes were correlated with changes in the content of other important aroma compounds to find specific structural genes that may be important for biosynthesis of these volatiles (Supplementary Fig. S3). For example, it was found that the genes *LOX*, *ADH*, *CAD*, *HPL* and *BAHD* may be involved in the synthesis of hexyl acetate, which is an important VOC in *P. mume*.

### Analysis of proposed genes associated with biosynthesis of known floral volatiles

**Fig. 7:**
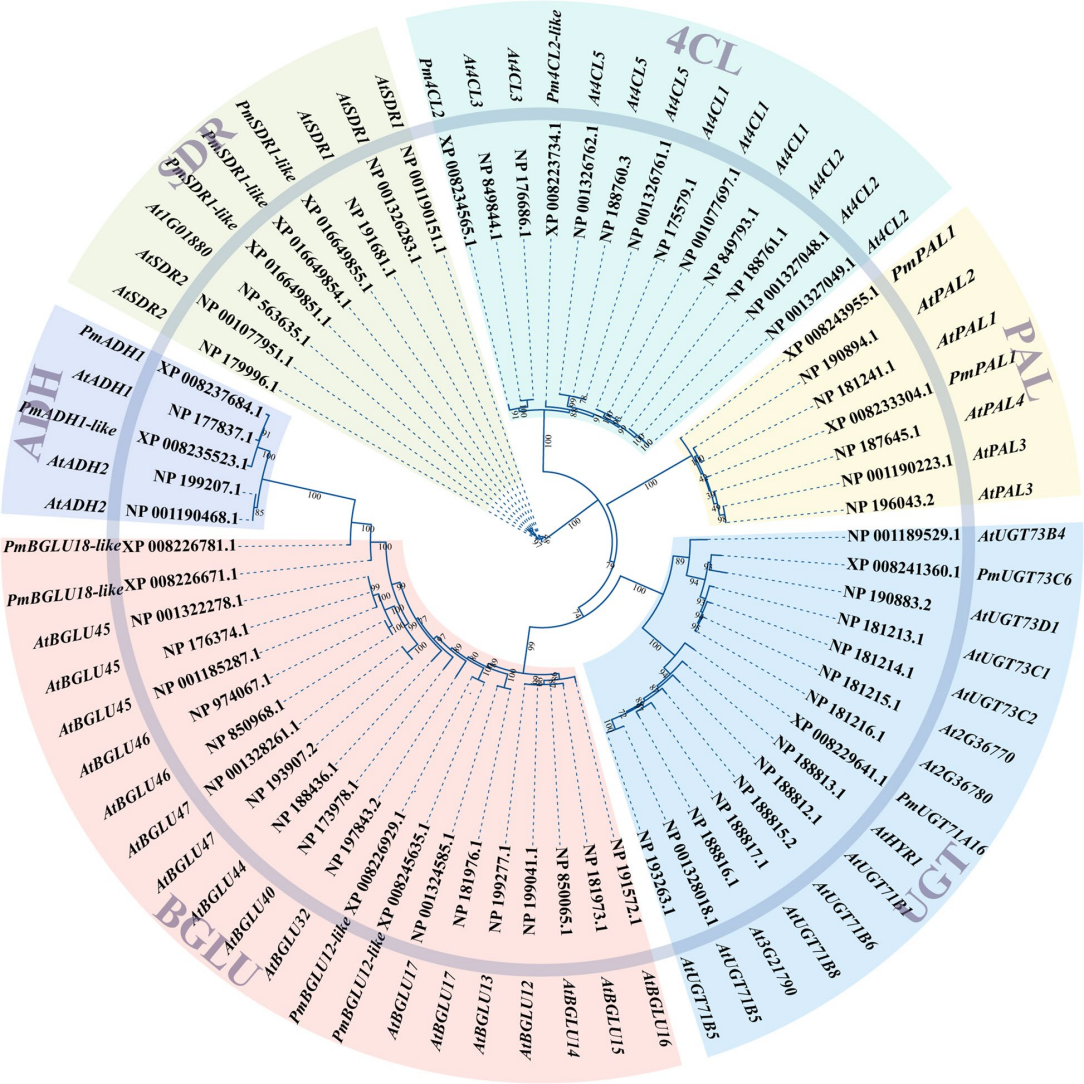
Proteins corresponding to genes highly relevant to the biosynthesis of floral aroma compounds in *Prunus* were screened by nucleotide sequence alignment with the related *Arabidopsis* proteins

Combining the results of GO enrichment, KEGG PATHWAY enrichment and WGCNA analysis of DEGs, a focused set of 101 genes was obtained that may be directly involved in the biosynthesis of *P. mume* aroma compounds. To specifically address the biosynthetic pathways of cinnamyl acetate, benzyl acetate and (iso)eugenol—and find the genes that play essential roles in these pathways in *P. mume*—the key coding enzymes were searched using the phmmer functionality of HMMER 3.0. The biosynthesis of benzene-containing aroma compounds in plants usually starts from shikimic acid, which is converted to phenylalanine through the shikimate pathway (involving aromatisation); a process that crosses the boundary of primary and secondary metabolism. Here, a phylogenetic tree was constructed by comparing the protein sequences of putative essential genes for *P. mume* fragrance formation with their verified homologues in *Arabidopsis thaliana* (Fig. 7). The results showed that the two *P. mume ADH* (*PmADH*) genes and two reported *A. thaliana ADH* (*AtADH*) genes fall into the same branch; that the three *PmSDR1-like* and *AtSDR1* protein sequences are very similar; and that the sequence similarity between *Pm4CL2* and *At4CL3* is high. Also, *PAL* genes were generally found to have high sequence homology between *Prunus* and *Arabidopsis* species. The genes *PmUGT73C6-like* and *AtUGT73D* clustered in one branch, whereas *PmBGLU18-like* clustered with the *AtBGLU45* and *AtBGLU46* genes, whose relationship to flower aroma volatilisation has been confirmed in *Arabidopsis* [27].

Phenylalanine ammonia lyase (PAL) catalyses the deamination of phenylalanine to *trans*-cinnamic acid, the primary and committed step of phenylpropanoid biosynthesis. There are two *PmPAL* genes (ncbi_103332355 and ncbi_103342143), with significant differences in their comparative transcriptome (Table S5). Furthermore, utilisation of phenylalanine is divided between two different pathways to synthesise phenylalanine-related compounds or other benzene compounds. We used the characterised *A. thaliana* genes from these pathways as bait to search the *P. mume* genome and found three *PmCAD*, two *Pm4CL*, two *PmCCR*, two *PmADH*, four *PmSDR* and three *PmCYP* genes (Table S5). Among these, the expression pattern of *PmCCR* was highly positively correlated with the content of volatile benzene compounds, and the expression of *PmCCR1* in LE was significantly higher than in GF.

β-Glucosidase (BGLU) is known to be widely present in various plant tissues. Its role is mainly in the prehydrolysis of aroma compounds, converting aromatic substances in the plant body from the bound (glycosylated) form to the free state and therefore releasing the aroma volatiles. In contrast, UDP-glycosyltransferase (UGT) acts in the opposite direction. The present transcriptomic data revealed that, in total, four β-glucosidases (*PmBGLU*) and two UDP-glycosyltransferases (*PmUGT*) were significantly differentially expressed in LE and GF (Table S5). The expression of all six of these genes was significantly upregulated in GF, relative to LE, at the S3 stage.

**Fig. 8:**
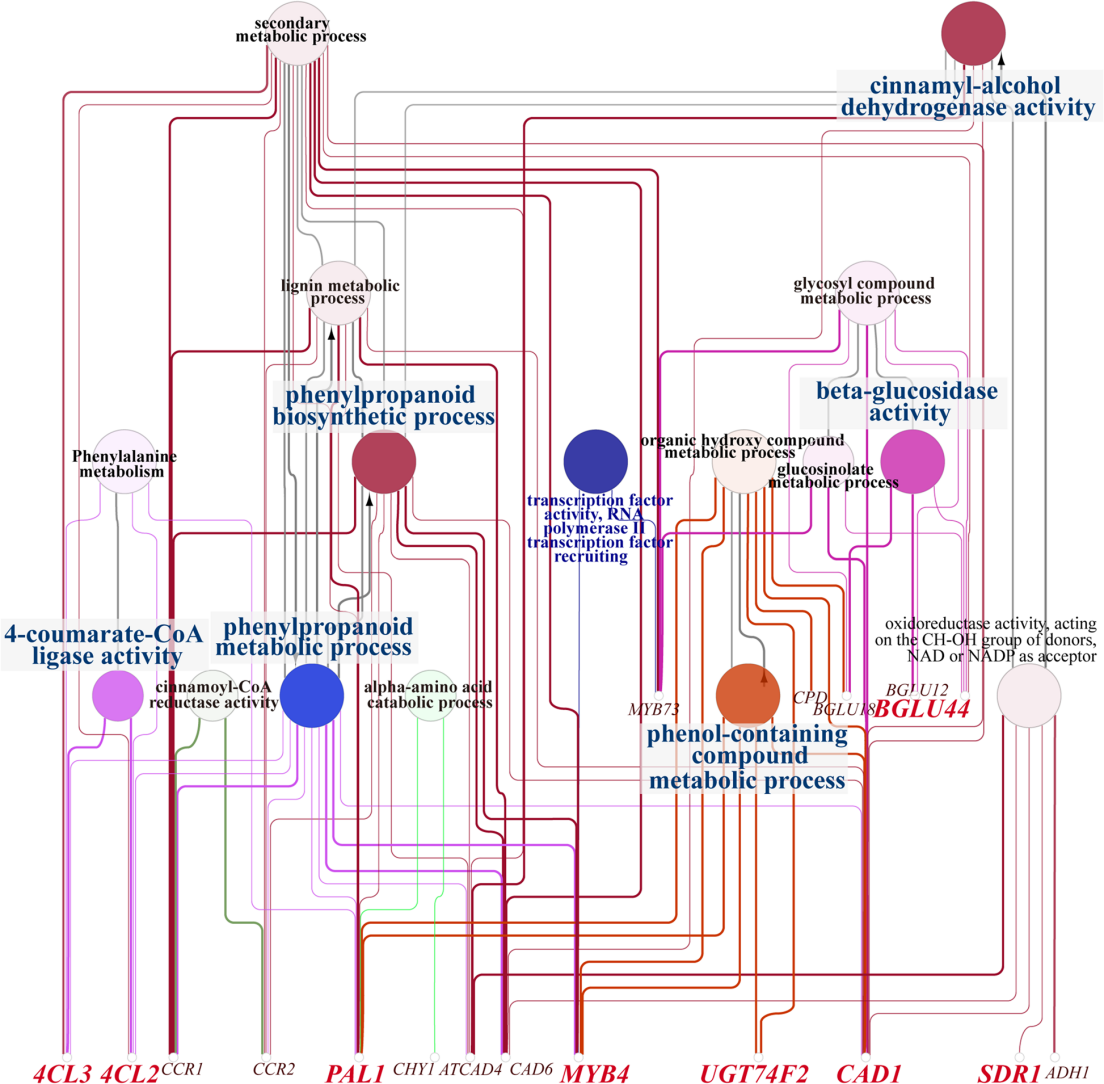
The key genes screened out by combining GO enrichment, KEGG PATHWAY enrichment [28] and WGCNA analysis were further analysed using Cytoscape. Nine key genes and *MYB* TFs were significantly enriched in a total of 18 metabolic processes

Further analysis of the enrichment network of these genes using the Cytoscape revealed that *4CL* and *CCR* were mainly enriched in upstream floral aroma synthesis processes, including phenylpropanoid metabolism, cinnamoyl-CoA reductase (CCR) activity and 4-coumarate: coenzyme A ligase (4CL) activity (Fig. 8). Both *PAL* and *CAD* (encoding cinnamyl alcohol dehydrogenase) were clearly enriched in a wider range of processes, including phenylpropanoid metabolism, phenylpropanoid biosynthetic processes, organic hydroxy compound metabolism and glucosinolate metabolism. In addition, *UGT* and *BGLU* were mainly enriched in the downstream stages of floral compound synthesis, in key functions of floral aroma formation such as phenol-containing compound metabolic processes and β-glucosidase activity. Taking a more general view, GO annotation of the genes and TFs identified during this analysis is anticipated to help determine possible relationships between key genes in the biosynthesis of *P. mume* aroma compounds and the molecular mechanisms of floral aroma compound synthesis and volatilisation.

### Exploration of key transcription factors

Transcription factors are thought to be critical for the proper control of many secondary metabolic processes. In our study of the comparative transcriptome of *P. mume*, 613 TFs (including transcriptional regulators) were identified from 76 families classified in the PlantTFDB database. On constructing reciprocal networks of differentially expressed TFs together with key genes (Fig. 8), we were surprised to find that *MYB4* transcription factors were enriched in phenol-containing compound metabolism, organic hydroxy compound metabolism, phenylpropanoid biosynthesis and phenylpropanoid metabolism, suggesting that these TFs might be important for regulation of the biosynthesis of volatile compounds [29]. In support of this possibility, *PmMYB4* TFs were upregulated in GF. Furthermore, *MYB73* was significantly enriched in glucosinolate metabolism and glycosyl compound metabolism, with *PmMYB73* transcriptionally downregulated in LE but consistently upregulated in GF.

**Fig. 9:**
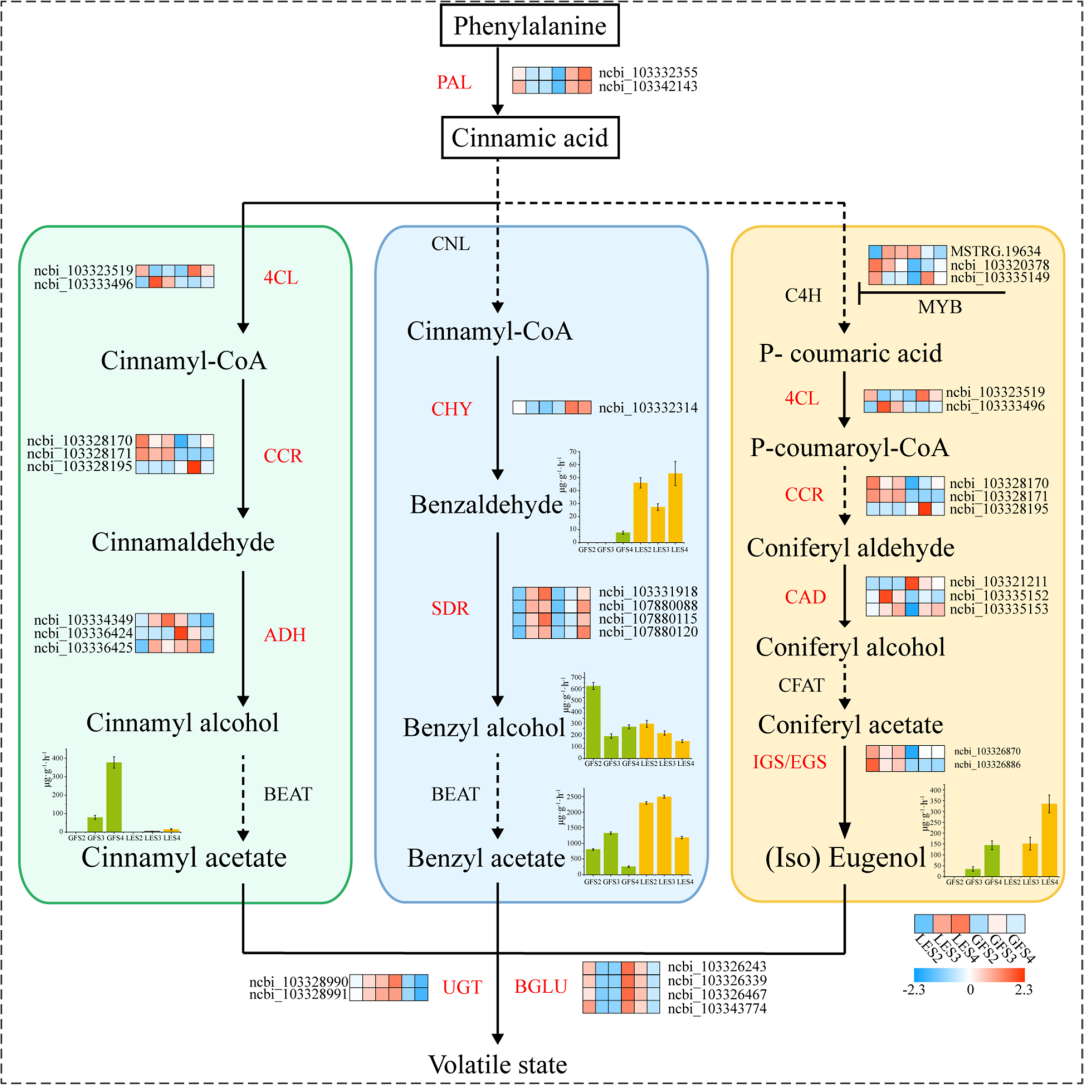
Putative regulatory model between key enes and TFs related to the biosynthesis of floral compounds in GF and LE petals. Enzyme names, unigene IDs and expression patterns are indicated for each step. The expression pattern of each unigene is represented by a grid of six squares. From left to right, the first three squares represent the relative log_2_(expression ratio) at the S2, S3 and S4 stages for LE; and the latter three squares represent the equivalent values for GF. The colour scale corresponds to log_2_(expression ratio), as indicated

Given this discovery of the involvement of core TFs, especially the *MYB* family, in VOC synthesis, the mechanisms of floral aroma formation in *P. mume* were interrogated further. Combining the observations regarding TFs with comparison of structural genes with verified protein function, we constructed a potential gene regulatory network for floral fragrance composition (Fig. 9), laying a foundation for uncovering the biosynthetic pathways of benzene compounds in LE and GF; especially cinnamyl acetate, benzyl acetate and eugenol.

### Gene expression validation

**Fig. 10:**
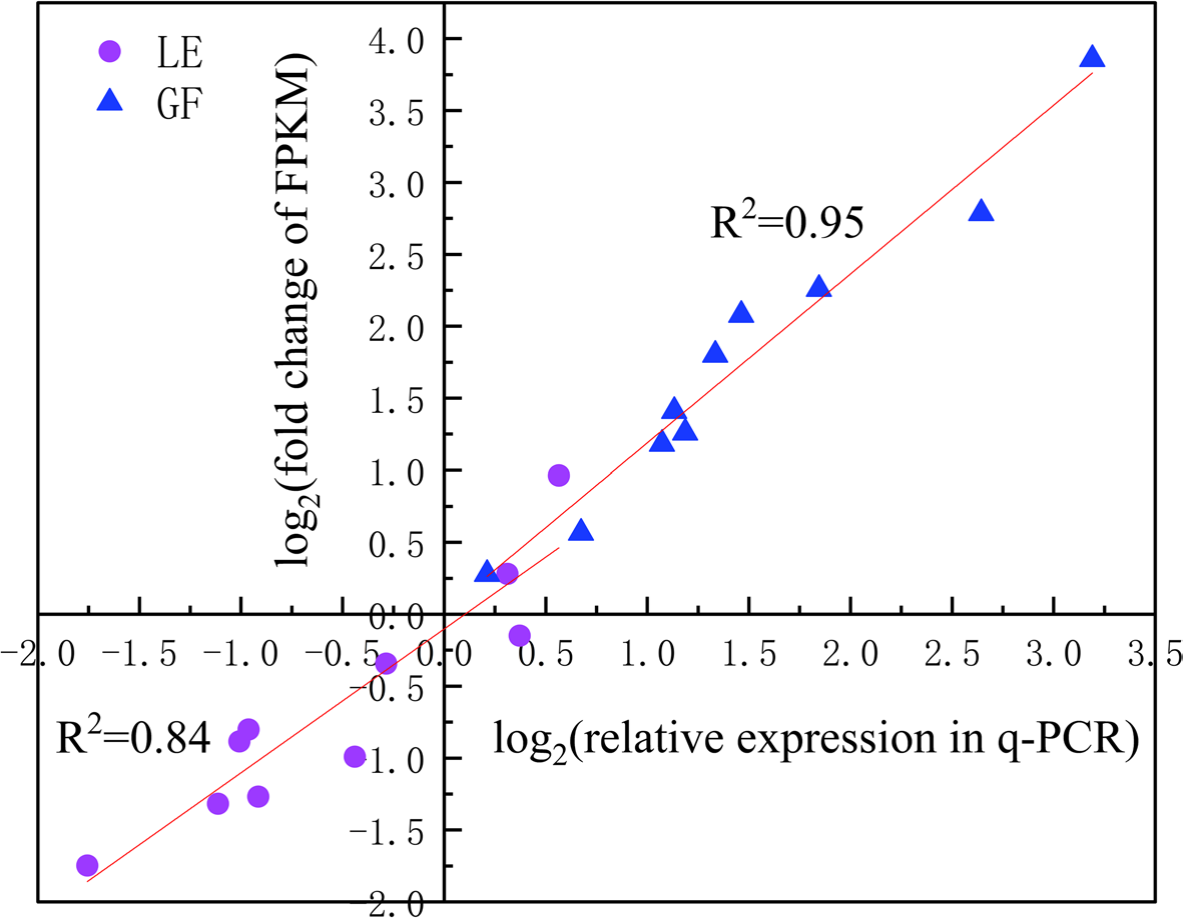
qRT-PCR validation of genes related to flower aroma compound biosynthesis in GF and LE petals at all three flowering stages

To confirm the integrity of the RNA-Seq results, we selected five key genes in the metabolic pathway of floral aroma compounds and one housekeeping gene (*PP2A*), and analysed relative expression levels in GF and LE by qRT-PCR for samples collected at the three flowering periods (Fig. 10). Pearson correlation analysis was used to evaluate the relationship between the RNA-Seq and qRT-PCR results for the different developmental stages (S2, S3 and S4). From a plot of log_2_(fold change in FPKM) from RNA-Seq against log_2_(2^−ΔΔCq^) from qRT-PCR, Pearson correlation coefficients of 0.95 and 0.84 were obtained for GF and LE, respectively, indicating that the RNA-Seq data were reliable.

## Discussion

### Dynamics of the main floral compounds of GF and LE

Floral fragrance is an important characteristic of ornamental plants, and the key components of this aroma consist of diverse secondary metabolites. The relative composition, amounts and interactions of these volatile compounds determine the specific floral fragrance [8], which is characterised by a high odour value (content/olfactory threshold) [30]. In the current study, we used GC-MS for relative quantification of 44 major volatile metabolites of *P. mume* and performed absolute quantification against external standards for six VOCs that were present at widely varying relative concentrations. The main volatiles identified were benzene compounds, with benzyl alcohol and benzaldehyde in particular being important precursors for the formation of floral aroma compounds typical of *P. mume* [13, 18]*.* The main components of its distinctive aroma are aromatics (eugenol, cinnamyl acetate and benzyl acetate) [31], and similarly, the floral aromas of many plants contain aromatic esters synthesised from the corresponding aromatic alcohols and acyl donors through reactions catalysed by acyltransferases [32, 33]. The amount of benzyl acetate released was higher for LE than for GF at all three flowering stages (Fig. 1c), and the odour threshold was lower, with an aroma of fruit and jasmine [31]. The amounts of benzaldehyde (with a sweet flower and fruity aroma) and eugenol (with a spicy clove odour) released by LE were also higher than for GF, and the interaction of these three volatiles may therefore explain why the aroma of the former is more sweet and fruity than the latter. In contrast, in the late flowering stage of GF, more cinnamyl acetate, cinnamyl alcohol and benzyl alcohol were detected, which manifest themselves with creamy and sweet aroma, and altogether shed the aroma around to be richer, stronger, and more intense. The biosynthesis of all of these floral aroma compounds was mainly influenced by expression of relevant genes in the biosynthetic pathways, enzyme activity, and substrate utilisation. The diversity of volatile aroma compounds and the abundance of *P. mume* germplasm therefore provide a material basis for the development of a wide range of aroma products.

### Key regulatory genes in the biosynthesis of cinnamic acid

As mentioned above, PAL is the first and key enzyme of the phenylpropanoid metabolic sequence, and is involved in secondary metabolic pathways in higher plants [34]. Phenylalanine produced via the shikimate pathway is deaminated by PAL to produce *trans*-cinnamic acid, which then enters the phenylpropanoid pathway [35]. In this work, comparative transcriptomic analysis revealed that two *PmPAL* genes were significantly more expressed in LE than in GF during S2, and vice versa during S4 (Table S6). High expression of *PmPAL* in LE during the S2 period would produce more *trans*-cinnamic acid, a precursor for the synthesis of volatile compounds, and may therefore explain why considerably greater amounts of VOCs were measured for LE than for GF at this stage (Fig. 1c).

A small number of plant TFs have previously been associated with the formation of floral fragrance. Petunia has four MYB members that regulate benzenoid volatiles [36], and by overexpressing the *MYB* genes from *A. thaliana* in petunia flowers, it was demonstrated that regulating the flow of precursors to phenylpropanoid metabolism affects volatile synthesis [37]. The MYB-like transcription factor *PhMYB* found in petunia indirectly inhibits the synthesis of *p*-coumaric acid derivatives by repressing the upstream enzyme, cinnamate-4-hydroxylase (C4H) [21]. Analysis of our transcriptomic data identified a *PmMYB4* gene (Table S6) that was significantly more expressed in GF than in LE, and was enriched in the metabolic processes of phenolic-containing compound metabolism and phenylpropanoid biosynthesis. These observations suggest that *PmMYB4* is also likely an important TF in *P. mume*, responsible for the significant differences in floral aroma composition between cultivars. Further exploration of the regulatory role of TFs in the synthesis of plum volatiles is therefore required, if the molecular mechanisms involved in biosynthesis of these aroma compounds are to be fully elucidated.

### Key genes for the induction of cinnamyl acetate and eugenol biosynthesis

The enzyme 4-coumarate: coenzyme A ligase (4CL) catalyses the third and final step of the phenylpropanoid metabolic pathway. It thus occupies a central position in the plant metabolic network, playing an important role in the biosynthesis of eugenol and cinnamyl acetate [38]. Although long explored, the biosynthetic pathway of benzaldehyde is still unresolved, despite it being a key intermediate in the synthesis of benzenoid compounds in *P. mume* aroma volatiles. In petunia, isotopic labelling was used to establish that benzaldehyde is derived from phenylalanine [39]; however, the benzenoid profile was unaffected if expression of *Ph4CL* gene was inhibited, suggesting that *Ph4CL* is not a major factor in the regulation of benzyl acetate biosynthesis in this species [40]. As stated above, and exemplified by previous studies, 4CL is an entry and control point for the phenylpropanoid and terpenoid biosynthetic pathways [41]; for example, its protein and mRNA expression profiles in basil were shown to be consistent with the production of phenylpropanoids and terpenoids in isolated glandular capillaries [42]. Here, correlation analysis between our comparative transcriptomic data and the aroma compound assay results revealed that two *Pm4CL* genes were significantly more highly expressed in LE than in GF, meaning they are likely involved in the synthesis of the volatile compounds cinnamyl acetate and eugenol.

Cinnamyl alcohol dehydrogenase (CAD) catalyses the conversion of cinnamaldehydes to the corresponding cinnamyl alcohol via an NADPH-dependent redox reaction [43], which is also the final step in the biosynthesis of lignin precursors [44]. Interestingly, the functions of different CAD protein members of the same species differ considerably [45]. Two CADs have been characterised in *A. thaliana*, AtCAD4 and AtCAD5, which show different substrate specificities but with the latter usually catalysing the synthesis of lignin monomers [46]. Comparison of the differential expression of *CAD* genes in LE and GF identified three key genes (Table S6) that might be involved in the synthesis of aroma compounds in *P. mume*. Correlating the transcriptomic data with the results of the aroma compound assay, it was hypothesised that *PmCAD1*, which is highly expressed in LE, is more likely to be involved in the synthesis of cinnamyl alcohol.

Biosynthesis of the phenylpropanoids eugenol and isoeugenol shares the same pathway as G-type lignin monomers in the early stages, up until the lignin monomer coniferyl alcohol is produced by CAD, at which point the phenylpropanoid pathway diverges from the lignin pathway [47]. Subsequent esterification produces coniferyl acetate, which is then converted by either of two enzymes—eugenol synthase (EGS) or isoeugenol synthase (IGS)—into eugenol or isoeugenol, respectively, which essentially belong to the same class of compounds [25]. Current studies on the molecular aspects of these floral volatiles have focused on the final step of (iso) eugenol anabolism, with 18 *EGS* and *IGS* genes having been identified in 10 plants including basil and petunia [48–51]. Analysis of the transcriptomic data obtained in this work revealed two genes annotated as isoeugenol synthases that were both expressed in LE at higher levels than in GF, consistent with the observed trend in metabolites and suggesting that these two *PmIGS* genes (Table S6) are required for the final step of isoeugenol biosynthesis.

### Key genes inducing the biosynthesis of benzyl acetate

Short-chain dehydrogenase/reductase (*SDR*) genes are involved in many aspects of primary and secondary metabolism and are notable in carbonyl–alcohol redox [52]. The *SDR* family is important for biosynthesis of some compounds derived from phenylalanine, such as in *Rosa damascena* flowers, where 2-phenylethanol (2PE) is synthesised from phenylalanine via phenylacetaldehyde as an intermediate. Reduction of the latter to 2-PE is catalysed by phenylacetaldehyde reductase (PAR), an SDR family member [53]. In this study, four *PmSDR* genes were detected in the transcriptomes of LE and GF (Table S6) and found to be involved in the biosynthesis of benzyl acetate. Their expression was consistently upregulated, with more benzyl alcohol detected in both cultivars during S4. Significant expression of *PmPAL* and *PmCNL* genes was detected during the benzaldehyde production phase in a recent study of *P. mume*, suggesting that endogenous benzaldehyde might originate from two pathways [25]. In *P. mume*, two P450s were coexpressed to produce the cyanogenic glycosides prunasin and amygdalin, which are derived from phenylalanine with benzaldehyde implied as a biosynthetic intermediate. The plant cytochrome P450 gene family catalyses a large number of mono-oxidation/hydroxylation reactions in plant primary and secondary metabolism [54], and thus, as an integral part of the benzenoid pathway, P450s might also have an important influence on the benzaldehyde synthesis pathway [27]. An in-depth study of the *SDR* and *CYP* gene families in *P. mume* may therefore help unravel the synthesis of benzyl acetate and the accumulation of benzyl alcohol as it occurs in these species.

### Key genes affecting the volatilisation of aroma compounds

Glycosylation of plant small molecules is a very common physiological phenomenon, and is one of the main mechanisms by which plant cells maintain metabolic homeostasis [55]. Previous studies on the *UGT* gene family have focused on its involvement in plant biotic stress response [56]. By comparative transcriptomic analysis, we found three *UGT* genes (*PmUGT71A16*, *PmUGT73C6*, *PmUGT74F2*) with potential involvement in the biosynthesis and volatilisation of *P. mume* aroma compounds. Of these, *PmUGT74F2* was significantly enriched in phenol-containing compound metabolism and organic hydroxy compound metabolism, suggesting its involvement in regulation of the volatilisation of *P. mume* aroma compounds.

A total of five DEGs annotated as β-glucosidases were detected in LE and GF (Table S6), all showing downregulation as flowering development progressed, and with expression in GF significantly higher than that in LE. β-Glucosidases are found in a wide range of plant tissues and, in contrast to UGTs, act mainly in the prehydrolysis of aroma compounds, converting aromatic substances in the plant from the bound to the free state and releasing aroma volatiles [57, 58]. Hydrolysis of cinnamon using β-glucosidase was found to release ‘hidden’ aroma compounds and to significantly increase the aroma concentration [27]. Two *A. thaliana* glucosidases, AtBGLU45 and AtBGLU46, clustered together with *Pinus contorta* β-glucosidase, and phylogenetic analysis suggested they were all able to hydrolyse eugenol and cinnamyl glycosides [59]. It is interesting to note that although the aroma of *P. mume* gradually became more intense from stages S2 to S4—and the aroma volatile content was consistently higher in LE than in GF—the *PmBGLU* gene was progressively downregulated in both cultivars over the three development stages, and it was expressed significantly more in GF than in LE. These observations may reflect a series of upstream processes that synthesise more aroma compounds in LE, making more substrates for prehydrolysis reactions. In addition, we found that *PmUGT71A16* was continually upregulated in LE and downregulated in GF, implying glycosylation of more of the aroma compounds produced in GF, and thus lower levels of these compounds in the volatile state. This may be a major reason why more aroma compounds were released from LE than GF, and it is also speculated that glycosylation takes priority over prehydrolysis in *P. mume*.

## Conclusion

In this study, OPLS-DA and OAV analyses identified six main floral aroma components for the two *P. mume* cultivars across three development stages: benzyl acetate, eugenol, benzaldehyde, benzyl alcohol, hexyl acetate and cinnamyl acetate. This composition of VOCs may define the specific aroma characteristic of *P. mume*. On combining these volatile compound measurements with transcriptome analysis, we found that the *PmPAL* enzymes and *PmMYB4* TFs may play prominent roles in regulating the accumulation of key precursor substrates (*trans*-cinnamic acid and *p*-coumaric acid derivatives) of the major *P. mume* aroma compounds. Furthermore, we obtained evidence supporting involvement of *PmCYP* genes and *PmSDRs* in controlling the biosynthesis of some key precursors, such as benzaldehyde and benzyl alcohol, in the benzyl acetate synthesis pathway. Association analysis of transcriptomic data and the key aroma substances revealed that three *CAD* genes, two *4CL* genes, three *CCR* genes and two *IGS* genes are likely to be important for synthesis of cinnamyl acetate and isoeugenol. Finally, the *PmUGT71A16* and *PmUGT73C6* genes are proposed as key factors controlling the morphology (bound and volatile states) of aroma compounds. These results have established a practical basis for further exploration of the regulatory mechanisms underlying the formation of aroma compounds in *P. mume*.

## Methods

### Plant materials

Two *P. mume* cultivars with different flower fragrances, ‘Xiao Lve’ (LE) and ‘Xiangxue Gongfen’ (GF), were chosen as the experimental materials. The two *P. mume* cultivars are not wild plants; instead, we have collected the plant materials in the Mei Garden of Jiufeng National Forest Park (39°54′ N, 116°28′ E) from March to April 2019. We have also obtained specific permissions regarding our collections and experiments with respect to institutional approval. The flowers were selected in advance from the upper part of the branches at 10:00 am on a clear day, and specimens at three stages of flowering (open petal, first bloom and full bloom) were selected as samples, cut off, then wrapped in aluminium foil and frozen in liquid nitrogen until testing of the petals. Transcriptome sequencing analysis (RNA-Seq) was performed on samples corresponding to the petal baring (S2), early flowering (S3) and full flowering (S4) periods of LE and GF (Fig. 1a). Each experiment was run in triplicate, making a total of 18 samples analysed, and the mean value of replicates was taken by default for subsequent analyses.

### Floral scent emission and quantitative analysis

For both GF and LE, three intact fresh flowers were selected for each developmental stage. Only the petals were picked, and those from each individual sample were placed in a 15 mL well-sealed headspace vial and left to stand for 30 min. A 2 cm solid-phase micro-extraction (SPME) fibre coated with divinylbenzene/carboxane/polydimethylsiloxane (50/30 μm DVB/CAR/PDMS) was selected to collect the volatiles. The extraction head was first placed in the sample inlet of a Shimadzu GCMS-QP2010 (Shimadzu, Kyoto, Japan) coupler and aged at 250 °C for 3 min. The extraction head was then inserted into a sample headspace vial and placed in a water bath at 30 °C for 30 min in the headspace. Then, the SPME fibre was inserted into the GC injection port for desorption (5 min, 250 °C) and subsequent analysis. GC-MS analysis was carried out using the Shimadzu QP2010 instrument equipped with a DB-5 ms quartz capillary column (30 m × 0.25 mm, 0.25 μm film thickness; Shimadzu, Kyoto, Japan). Run parameters were: carrier gas purity 99.999%, inlet temperature 250 °C, split injection, total flow rate 27.2 mL min^− 1^, split ratio 20, ion source temperature 200 °C and interface temperature 250 °C. The total run time was 36.17 min with an initial temperature of 50 °C, held for 1 min then ramped up to 120 °C at 5 °C min^− 1^, then to 200 °C at 8 °C min^− 1^, then to 250 °C at 12 °C min^− 1^ and held for 7 min. The electron potential of the mass spectrometer was set to 1 kV and the mass scan range was *m/z* 30–500 in full scan mode.

Unknown compounds were identified by comparison with the NIST11 library (National Institute of Standards and Technology 2011, Shimadzu, Japan), in combination with manual profiling to determine the individual chemical components. The main volatile compounds were quantified by external standard methods [31]. Standards of benzaldehyde, benzyl alcohol, eugenol, benzyl acetate, hexyl acetate and cinnamyl acetate (concentration ≥ 98%) were serially diluted, using methanol (chromatographically pure) as the dilution solvent, to give five-step concentration gradients of 0.05, 0.1, 0.25, 0.5 and 0.8 μg g^− 1^. For each reference solution, a 1 μL aliquot was analysed using the same GC-MS conditions as for the volatile compounds. A standard curve was plotted based on the peak area and concentration of the standards, then a linear regression model was used for quantitative analysis of the same volatile components in the test samples. The OAV of a compound was calculated by dividing the calculated volatilisation with established sensory thresholds, in accordance with the literature [60–62]. Odour descriptions were also obtained from the literature [63].

### RNA-Seq and data processing

RNA extraction was performed using the Trizol kit (Invitrogen, Carlsbad, CA, USA). RNA degradation and contamination were then analysed using agarose gel electrophoresis, and RNA purity was assessed using an Agilent 2100 Bioanalyzer.

After the RNA samples were verified for quality, eukaryotic mRNA with polyA tails was enriched using oligo (dT) magnetic beads, while a Ribo-Zero™ Magnetic Kit (Bacteria) (Epicentre, Madison, WI, USA) was used to further enrich the mRNA by removing prokaryotic rRNA. The enriched mRNA fragments were then converted into short fragments using fragmentation buffer, followed by random reverse transcription to cDNA. Second strand cDNA was synthesised using DNA polymerase I, RNase H, dNTPs and buffer. End repair was performed with the QiaQuick PCR extraction kit (Qiagen, Venlo, Netherlands), then poly(A) was added and the fragments were ligated to Illumina sequencing adapters. Amplification and sequencing were performed using an Illumina HiSeq 2500. As noted above, three biological replicates were prepared for each sample.

High quality reads were obtained by removing reads with connectors, those containing more than 10% unknown nucleotides (N), and those containing more than 50% low quality bases (Q value ≤20). Bowtie2 (version 2.2.8) was used to map read segments to the ribosomal RNA (rRNA) database [64], then rRNA-mapped reads were deleted. The remaining clean reads were used further for assembly and gene abundance calculation. Search and alignment to the reference genome were carried out with HISAT 2.2.4, using the “--rna-strandness” option with all other parameters set to default values [65].

### Data analysis

An OPLS-DA model was applied to discriminate between the two *P. mume* cultivars, using SIMCA 14.1. The DESeq (2012) R package was used for differential gene expression analysis. To identify DEGs, the following constraints were applied to pairwise comparisons: FPKM > 5, FDR < 0.01, log_2_ fold change > 1 or < − 1. The DESeq (2012) R software package based on hypergeometric distribution was used for principal component analysis (PCA) and gene ontology (GO) enrichment analysis of DEGs. Kyoto Encyclopedia of Genes and Genomes (KEGG) analysis was used to explore pathways of DEGs [28, 66]. WGCNA analysis was performed according to Langfelder and Horvath [67]. The interaction network was analysed by Cytoscape 3.8.2, and the protein sequences of *P. mume* (sequence data from https://www.ncbi.nlm.nih.gov/, version *P. mume*_V1.0) and *A. thaliana* (sequence data from https://www.arabidopsis.org/, version TAIR 10) were compared by MEGA7 (10.1093/molbev/msw054). Genetic conserved domains were searched in the EMBL-EBI database (http://pfam.xfam.org/). Sequence alignment was performed using the MUSCLE tool, and phylogenetic tree construction using the FastME tool. Adobe Illustrator CC 2021 was used for visual presentation of graphics.

### qRT-PCR validation

The real-time quantitative reverse transcription polymerase chain reaction (qRT-PCR) method was employed to detect the expression of six genes related to floral biosynthesis in the petals of LE and GF (Table S7), using the fluorescence-based quantitative PCR instrument, ABI Step One Plus (Applied Biosystems, USA). According to the manufacturer’s instructions provided with the reverse transcription kit (Novizan R223, China), 50 ng–2 μg total RNA was used to establish each reaction in a volume of 20 μL. Amplification proceeded as follows: predenaturation at 95 °C for 90 s, followed by 40 cycles of 95 °C for 5 s, 65 °C for 30 s (annealing), and 72 °C for 20 s. Dissociation curves were recorded from 65 to 95 °C. Each reaction was repeated three times. The relative expression levels of target genes were calculated using 2^−ΔΔCq^, and the data were analysed with SPSS 26.0 and Origin 8 software. The primer sequences used for qRT-PCR analysis are listed in Supplementary Table 7.

## Supplementary Information

**Additional file 1: Fig. S1.** KEGG pathway enrichment of the differential expressed genes at the three flowering stages of GF and LE. (|log2FC| > 1, FDR < 0.01). **Fig. S2.** Gene ontology pathway enrichment of the differential expressed genes at the three flowering stages of GF and LE. **Fig. S3.** Correlation analysis of other important volatile compounds with key genes. The colour of the heatmap ranges from purple (value, − 2.5) to yellow (value, + 2.5) on a natural logarithmic scale.

**Additional file 2: Table S1.** Identification of the aroma compound from the two *Prunus mume* cultivars. **Table S2.** Overview of the transcriptome sequencing dataset and mapping statistics from GF and LE petal samples. **Table S3.** Key DEGs identified by enrichment analysis at the different flowering stages, showing functional categories of significantly over-represented DEGs at S2, S3, S4 and in LE vs. GF. **Table S4.** The name of the pathway associated with each KEGG term. **Table S5.** List of 101 genes that may be directly involved in the biosynthesis of *P. mume* aroma compounds. **Table S6.** List of key regulatory genes in the biosynthesis of cinnamic acid, cinnamyl acetate, eugenol, benzyl acetate and key genes affecting the volatilisation of aroma compounds. **Table S7.** The primer sequences of genes used for quantitative real-time PCR. **Table S8.** Summary of samples information uploaded to NCBI.

## Data Availability

The datasets generated and/or analysed in the course of this study are available from the NCBI repository (BioProject ID: PRJNA786063) (https://www.ncbi.nlm.nih.gov/nuccore/)(Table S8) [68]. Public access to the databases mentioned above are open and no administrative permissions are needed for accessing and using the data. Material samples are available from authors.

## Abbreviations

PAL: Phenylalanine ammonia lyase
MYB: Myeloblastosis viral oncogene
CYP: Cytochrome P450
CAD: Cinnamyl alcohol dehydrogenase
4CL: 4-Coumarate:coenzyme A ligase
CCR: Cinnamoyl-CoA reductase
BGLU: β-Glucosidase
UGT: UDP-glycosyltransferase
ADH: Alcohol dehydrogenase
SDR: Short-chain dehydrogenase/reductase
TF: Transcription factor
C4H: Cinnamate-4-hydroxylase
CNL: Trans-cinnamate:coA ligase
EGS: Eugenol synthase
IGS: Isoeugenol synthase
