# Supplementary figures and images for: Comparative transcriptome analysis linked to key volatiles reveals molecular mechanisms of aroma compound biosynthesis in Prunus mume
Source: BMC Plant Biol. 2022 Aug 9;22:395. doi: 10.1186/s12870-022-03779-3 (PMC9361687; doi:10.1186/s12870-022-03779-3)

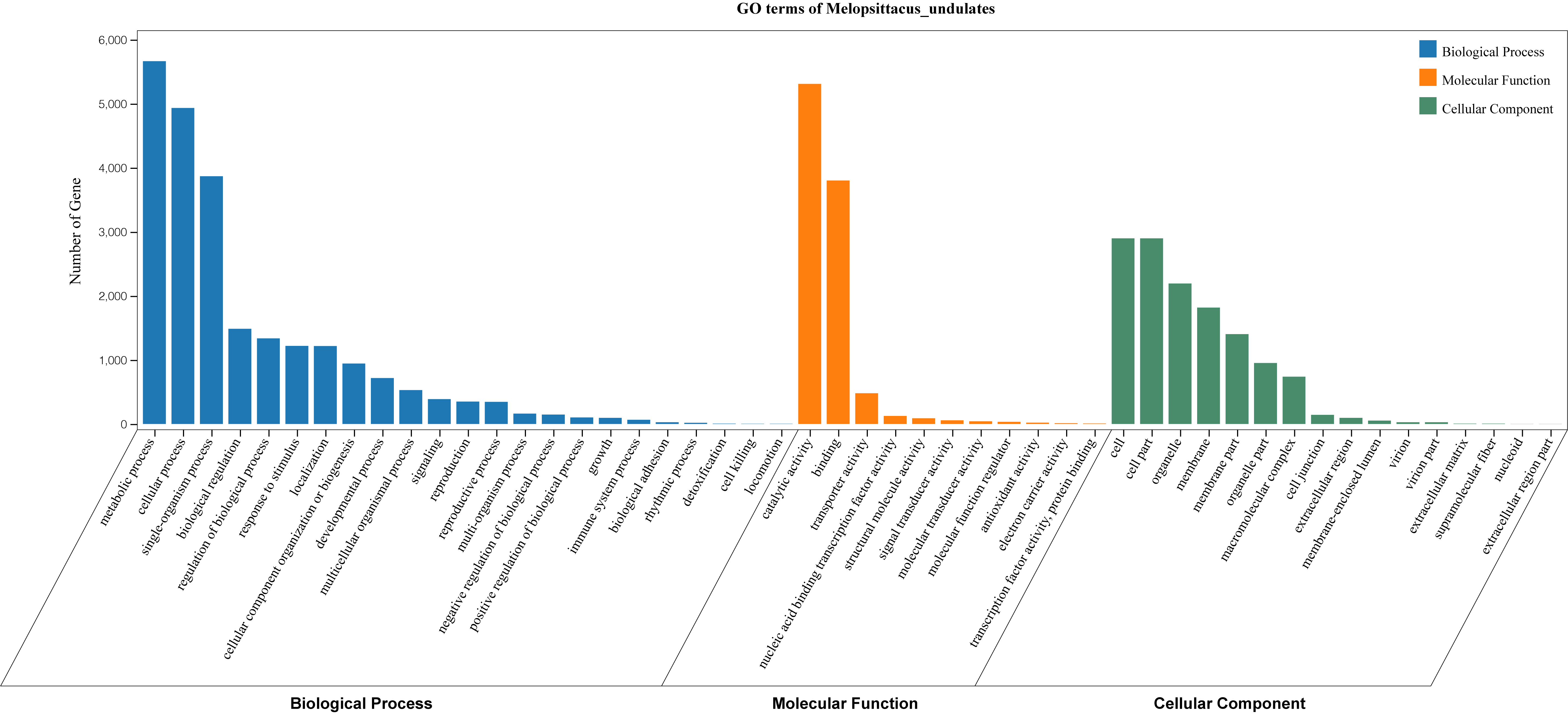

Supplement: Supplementary file 1 — Additional file 1: Fig. S1. KEGG pathway enrichment of the differential expressed genes at the three flowering stages of GF and LE. (|log2FC| > 1, FDR < 0.01). Fig. S2. Gene ontology pathway enrichment of the differential expressed genes at the three flowering stages of GF and LE. Fig. S3. Correlation analysis of other important volatile compounds with key genes. The colour of the heatmap ranges from purple (value, − 2.5) to yellow (value, + 2.5) on a natural logarithmic scale. [file 12870_2022_3779_MOESM1_ESM.zip › Figure S1.tif]

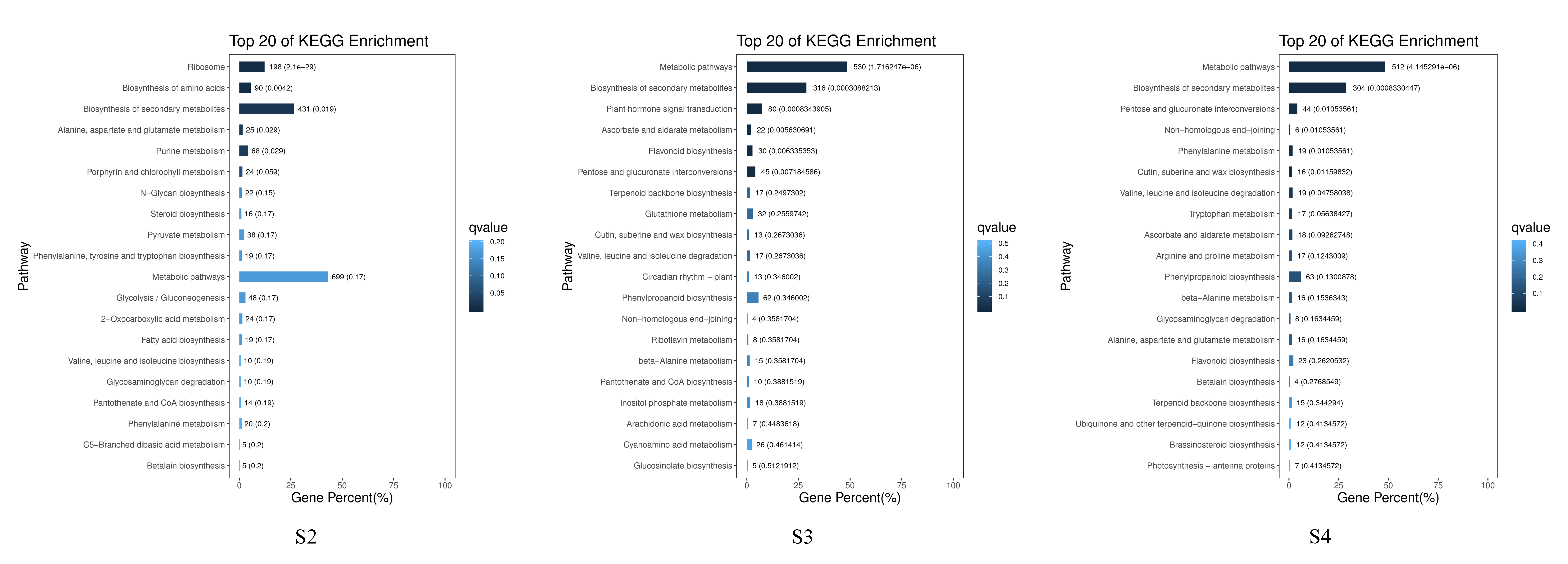

Supplement: Supplementary file 1 — Additional file 1: Fig. S1. KEGG pathway enrichment of the differential expressed genes at the three flowering stages of GF and LE. (|log2FC| > 1, FDR < 0.01). Fig. S2. Gene ontology pathway enrichment of the differential expressed genes at the three flowering stages of GF and LE. Fig. S3. Correlation analysis of other important volatile compounds with key genes. The colour of the heatmap ranges from purple (value, − 2.5) to yellow (value, + 2.5) on a natural logarithmic scale. [file 12870_2022_3779_MOESM1_ESM.zip › Figure S2.tif]

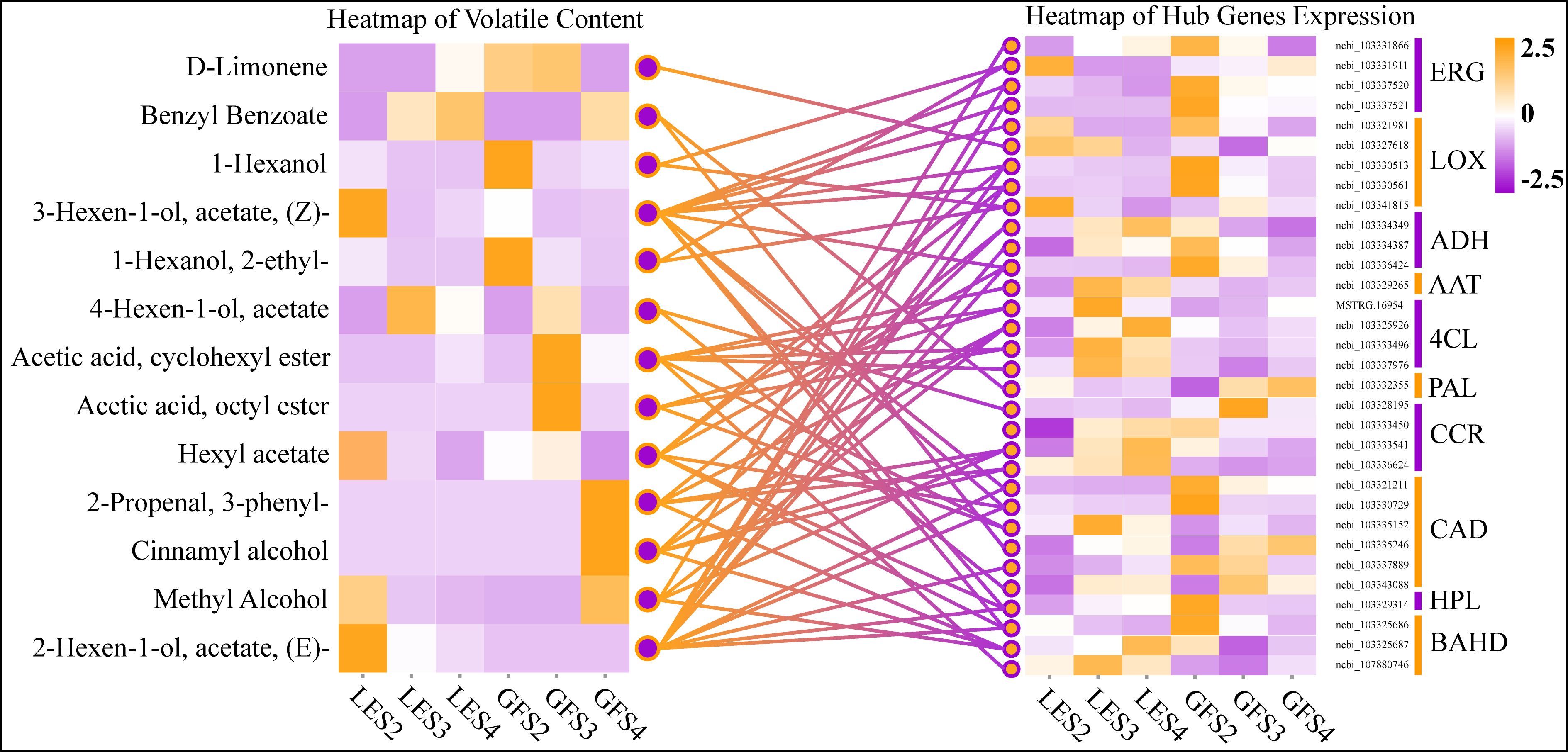

Supplement: Supplementary file 1 — Additional file 1: Fig. S1. KEGG pathway enrichment of the differential expressed genes at the three flowering stages of GF and LE. (|log2FC| > 1, FDR < 0.01). Fig. S2. Gene ontology pathway enrichment of the differential expressed genes at the three flowering stages of GF and LE. Fig. S3. Correlation analysis of other important volatile compounds with key genes. The colour of the heatmap ranges from purple (value, − 2.5) to yellow (value, + 2.5) on a natural logarithmic scale. [file 12870_2022_3779_MOESM1_ESM.zip › Figure S3.tif]
